# Relationship between gait quality measures and modular neuromuscular control parameters in chronic post-stroke individuals

**DOI:** 10.1186/s12984-021-00860-0

**Published:** 2021-04-07

**Authors:** Sung Yul Shin, Yusung Kim, Arun Jayaraman, Hyung-Soon Park

**Affiliations:** 1 Department of Mechanical Engineering, Korea Advanced Institute of Science and Technology (KAIST), Daehak-ro 291, Yuseong-gu, Daejeon, 34141 Republic of Korea; 2 Max Nader Lab for Rehabilitation Technologies and Outcomes Research, Shirley Ryan AbilityLab, 355 E Erie St, Chicago, IL 60611 USA; 3 Department of Physical Medicine and Rehabilitation, Northwestern University, 710 N Lake Shore Dr, Chicago, IL 60611 USA

**Keywords:** Muscle module, Muscle synergy, Gait quality, Gait symmetry, Stroke

## Abstract

**Background:**

Recent evidence suggests that disinhibition and/or hyperexcitation of the brainstem descending pathways and intraspinal motor network diffuse spastic synergistic activation patterns after stroke. This results in simplified or merged muscle sets (i.e., muscle modules or synergies) compared to non-impaired individuals and this leads to poor walking performance. However, the relations of how these neuromuscular deficits influence gait quality (e.g., symmetry or natural walking patterns) are still unclear. The objective of this exploratory study was to investigate the relations of modular neuromuscular framework and gait quality measures in chronic stroke individuals.

**Methods:**

Sixteen chronic post-stroke individuals participated in this study. Full lower body three-dimensional kinematics and electromyography (EMG) were concurrently measured during overground walking at a comfortable speed. We first examined changes in gait quality measures across the number of muscle modules using linear regression model. Then, a stepwise multiple regression was used to investigate the optimal combination of the neuromuscular parameters that associates with gait quality measures.

**Results:**

We observed that subjects who had a lower number of muscle modules revealed reduced function (i.e., speed) and greater asymmetry in the kinematic parameters including limb length, footpath area, knee flexion/extension, and hip abduction/adduction (all p < 0.05). We also found that the combination of input variables from the modular neuromuscular control framework significantly associated with gait quality measures (average ${R}^{2}=42.5\mathrm{\%}$). Those variables included variability accounted for ($VAF$) information from the muscle modules and area under the EMG envelope curves of the quadriceps (i.e., rectus femoris and vastus lateralis) and tibialis anterior muscles.

**Conclusions:**

The results suggest that there exists a significant correlation between the neuromuscular control framework and the gait quality measures. This study helps to understand the underlying mechanism of disturbances in gait quality and provides insight for a more comprehensive outcome measure to assess gait impairment after stroke.

**Supplementary Information:**

The online version contains supplementary material available at 10.1186/s12984-021-00860-0.

## Background

Effective gait recovery after a stroke involves improvements both in functional mobility and quality of movement. However, conventional clinical outcomes measuring gait such as Ten Meter Walk Test (10MWT), Six Minute Walk Test (6MWT), and Timed-Up-and-Go (TUG) focus on functional indices that provide a holistic picture of walking performance and recovery [1–3]. On the other hand, an increased number of recent studies accentuate the importance of monitoring detailed gait quality to assess gait impairments [4]. Disturbances in gait quality are associated with an increased risk of falls [5], greater energy expenditure [6], and long-term problems such as learned non-use or use-dependent plasticity, musculoskeletal injuries, and pain [7, 8].

Symmetry is a common measure to characterize disturbances in gait quality. While spatiotemporal symmetry (e.g., step length, step time) has been well charted to describe gait after stroke [9], kinematics would arguably be the most detailed way to represent the human movements including gait after stroke. Post-stroke individuals exhibit significant asymmetry in joint kinematics with greater inter-individual variability than spatiotemporal measures [10]. Typical asymmetry in joint kinematics includes reduced hip extension, knee flexion and ankle dorsi/plantar flexion, and knee hyperextension on the impaired side [11]. Limb kinematics, related to the end-effector (i.e., foot) motion in task space, has also been indicated as an important parameter for locomotor function [4]. For instance, Shin et al., found that post-stroke individuals preferentially coordinated the paretic side of limb function using limb kinematics by compensating joint kinematics during walking [4].

Neuromuscular activity is crucial to execute biomechanical functions such as gait [12]. Previous studies have shown that muscle activity during walking can be grouped into sets of co-excited muscles (also known as muscle modules or synergies) [13]. While there is an ongoing debate on whether these modules are originated from neural plasticity shaped by repetitive activities or encoded in central nervous system, a general consensus is that modules may reduce the computational cost in selecting strategies of motor coordination [14, 15]. Previous studies have identified that well-coordinated gait in healthy individuals can be produced by a small number between four to five group of modules [13, 16]. Other studies suggest that the concept of muscle modules can be used as an outcome measure to assess motor recovery following therapeutic interventions [17].

Recent evidence suggests that disinhibition and/or hyperexcitation of the brainstem descending pathways and intraspinal motor network diffuse spastic synergistic activation post-stroke [18]. As a result, simplified or merged muscle modules compared to non-impaired individuals are typically observed and lead to poor walking performance, for instance, reduced walking speed with greater spatiotemporal asymmetry than those of healthy individuals [13]. Several other previous studies discussed the impact of neuromuscular deficit on gait impairments or deviations after stroke [19, 20]. For example, Barroso et al. combined biomechanics and modular parameters to predict measures of walking asymmetry such as paretic limb propulsion or paretic stride ratio [20]. While these studies may provide multifaceted picture of walking performance and recovery, an additional endeavor to find how detailed kinematic gait quality measures are influenced by neuromuscular deficits, such as merged muscle modules, may help to better delineate the underlying causality among impairments and locomotor functions after stroke.

**Fig. 1:**
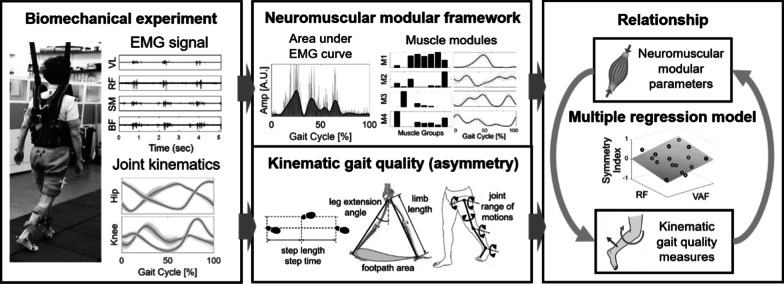
Overview of the study

The objective of this exploratory study was to investigate the relations of modular neuromuscular deficits and disturbances in gait quality measures (i.e., asymmetry) in terms of spatiotemporal, limb and joint kinematic parameters in chronic post-stroke individuals (see Fig. 1). We measured lower body electromyography (EMG) activities and gait kinematics concurrently during walking from 16 chronic post-stroke participants. We hypothesized that the post-stroke individuals with a reduced number of muscle modules will exhibit greater asymmetry in gait quality measures due to the loss of independence in motor activations [13]. Accordingly, we also expected to find a strong association between the gait quality measures and the input modular neuromuscular control framework assuming a causal relationship exists in these measures [21].

## Methods

### Participants

We recruited 16 individuals (6 left hemiparesis, 12 male, age: 62.9 $\pm$ 11.1 years) with chronic stroke (> 6 months) to participate in this study approved by the Institutional Review Board of Korea Advanced Institute of Science and Technology. Individuals ranged in age and impairment level (see Table 1). The inclusion criteria were as follows: at least 6 months after stroke, independent walking without falling regardless of walking speed, and over 70 points of Modified Barthel index. The exclusion criteria were as follows: perceptual and cognitive dysfunction and over 3 points on the Modified Ashworth Scale. Prior to the experiment, the experimenter explained all the experimental procedures to each participant and obtained informed consent.

**Table 1:** Demographics and clinical characteristics of the participants

ID	Age (years)	Sex	Height (cm)	Weight (kg)	Side affected	Time after stroke (years)	Type of lesion	FMA	FIM	Comfortable walking speed (m/s)
P1	59	M	170	69	R	4	Ischemic	21	11	0.29 $\pm$ 0.04
P2	61	M	169	72	L	3	Ischemic	16	9	0.27 $\pm$ 0.06
P3	70	M	158	69	R	2	Ischemic	12	12	0.16 $\pm$ 0.04
P4	55	F	158	60	L	11	Hemorrhage	22	14	0.24 $\pm$ 0.03
P5	59	M	176.5	66	R	3	Ischemic	13	8	0.22 $\pm$ 0.02
P6	73	M	170	74	Both	21	Hemorrhage	22	3	0.18 $\pm$ 0.02
P7	75	F	153	49	R	5	Ischemic	25	12	0.20 $\pm$ 0.02
P8	75	M	164	62	R	15	Ischemic	18	13	0.32 $\pm$ 0.04
P9	68	M	172	83	L	10	Ischemic	26	13	0.47 $\pm$ 0.02
P10	36	M	173	80	L	1	Ischemic	17	7	0.15 $\pm$ 0.03
P11	77	M	169	58	R	25	Ischemic	10	12	0.26 $\pm$ 0.01
P12	55	M	177	84	L	13	Ischemic	15	14	0.37 $\pm$ 0.03
P13	47	F	163	57	R	4	Hemorrhage	19	13	0.29 $\pm$ 0.04
P14	64	M	172	81	R	27	Ischemic	31	14	0.49 $\pm$ 0.04
P15	62	F	156	57	L	15	Hemorrhage	16	11	0.13 $\pm$ 0.03
P16	70	M	171	83	R	18	Ischemic	27	12	0.58 $\pm$ 0.03

*M* male, *F* female, *R* right, *L* left, *FMA* Fugl-Meyer assessment (max: 34), *FIM* Functional Independent Measure (locomotion, max: 14)

### Experimental setup and data collection

Full lower body three-dimensional kinematics and EMG data were collected concurrently during overground walking from each participant. Gait kinematics were acquired using the VICON Motion Capture System (MX T-series Vicon Motion Systems Ltd, Oxford, UK), consisting of eight cameras at 100 Hz. Prior to the EMG data acquisition, all participants underwent standard skin preparation including shaving and cleaning with alcohol to minimize skin impedance. Wireless surface EMG sensors from Delsys Trigno (Delsys, Inc., Natick, MA) were used to amplify and measure the electrical activity of muscles at the sampling rate of 2000 Hz. Biopolar EMG electrodes (Ag/AgCl) were placed on surface of the skin of 16 different muscles at bilateral thigh and lower leg identified by palpation. For example, to find the tibialis anterior, the subjects were asked to dorsiflex the ankle to activate the corresponding muscle while the experimenter pushed their instep to make an isometric condition. In this way, the location of each muscle on the unaffected side was palpated, and the muscles on the affected side were identified symmetrically. Those muscles included in this study were extensor halluces longus ($EHL$), tibialis anterior ($TA$), soleus ($SO$), gastrocnemius ($GA$), vastus lateralis ($VL$), rectus femoris ($RF$), semitendinosus ($SM$), and biceps femoris ($BF$).

Each participant completed 4–6 trials of walking back and forth on a plain six meters walkway at a comfortable speed. Participants did not use any of assistive devices such as a cane, walker or ankle foot orthosis during the recording sessions. All participants wore a harness without any body weight support for safety to catch them if needed to prevent possible falls.

### Kinematic data analysis

**Fig. 2:**
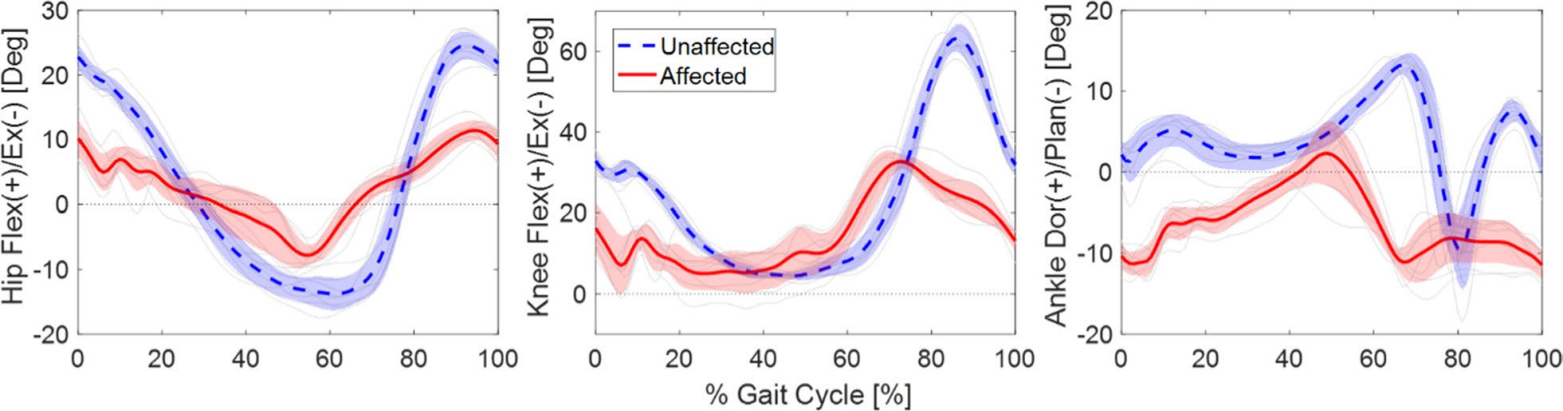
Example joint angle trajectories of a single gait cycle (heel strike to heel strike) in sagittal plane, hip flexion/extension (left), knee flexion/extension (middle), and ankle dorsi/plantar flexion (right)

Visual 3D v6 Professional (C-Motion, Inc., Germantown, MD) software was used to extract three-axis joint angle trajectories at pelvis and bilateral hip, knee and ankle from the marker data for each trial. Custom software was written in MATLAB (Mathworks, Inc. R2017b, Natick, MA) to calculate features and outcomes. The joint angle trajectories and EMG data of the gait portion were segmented from heel strike to heel strike events from each trial [22]. Each segmented gait cycle was normalized into 101 time points to represent 100% of the gait cycle using a cubic spline interpolation [23]. The average of joint trajectories in time normalized gait cycles were used as the representative joint trajectories of each subject. An example of the recorded gait kinematics data of bilateral joint angle trajectories of a single gait cycle in the sagittal plane is illustrated in Fig. 2.

### Modular neuromuscular control parameters

#### Area under the EMG envelope

Integrated EMG is defined as the area under the curve of the rectified EMG signal, which is one of the standard amplitude parameter to represent EMG signal characteristics [24]. It is a parameter that frequently used to compare EMG activation and is considered as a measure of voluntary muscle drive [25]. An increase in the integrated EMG signal period, amplitude, and power may represent an increase in firing frequency and a higher muscle fiber recruitment [26].

**Fig. 3:**
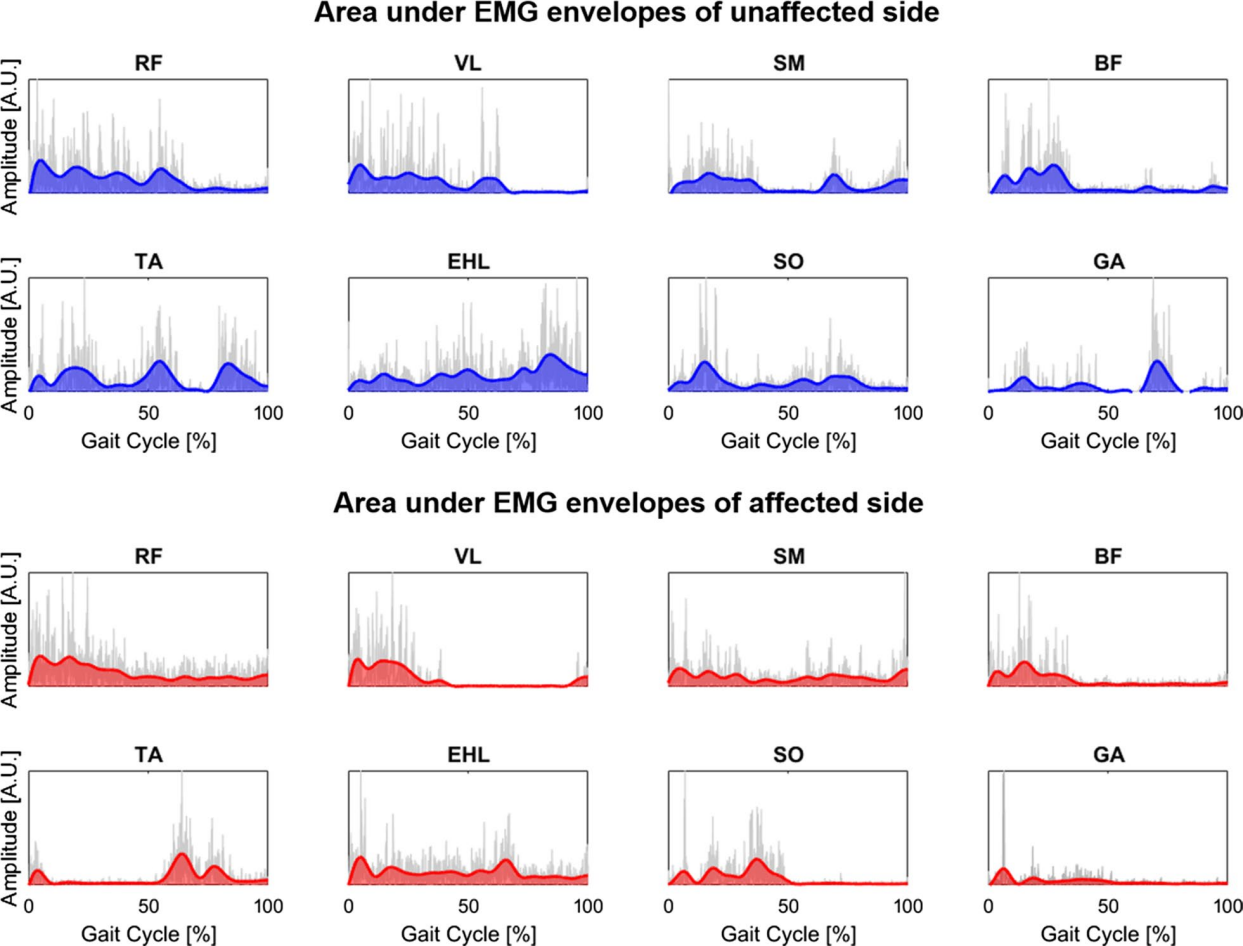
Example area under the EMG envelope curves of all 16 muscles of bilateral legs during a single gait cycle from participant P1 (grey lines are rectified EMG data; *A.U.*, arbitrary unit; $RF$, rectus femoris; $VL$, vastus lateralis; $SM$, semitendinosus; $BF$, biceps femoris; $TA$, tibialis anterior; $EHL$, extensor halluces longus; $SO$, soleus; $GA$, gastrocnemius)

The selected raw EMG signals from each participant were high-pass filtered at 40 Hz with a zero-lag fourth-order Butterworth filter, demeaned, rectified, and low-pass filtered with a zero-lag fourth-order Butterworth filter at 10 Hz, resulting in the EMG envelope [24]. For each muscle, the filtered signal was normalized to its peak value from across all gait cycles then the area under the EMG envelope curve was calculated and used to represent the neuromuscular indicator as ${A}_{i}$ where $A$ can be $EHL$, $TA$, $SO$, $GA$, $VL$, $RF$, $SM$, or $BF,$ and $i$ can be the unaffected $(US)$ or affected $(AS)$ side. An example area under the EMG envelopes of all 16 muscles used in the study from a participant (P1) is depicted in Fig. 3.

#### Muscle modules

The raw EMG signals were processed into EMG envelope patterns as previously described in [13]. Approximately nine (average 9.38 $\pm 3.2$) gait cycles per subject were included in the module analysis. For each subject, EMG envelope profiles of each gait cycle were normalized into 101 time points to represent 100% of gait cycle. The EMG profiles were combined into a $m\times t$ matrix (${EMG}_{o}$), where $m$ indicates the number of muscles and $t$ represents the time frame (i.e., number of gait cycles $\times$ 101). The EMG matrix, ${EMG}_{o}$, was decomposed into muscle group weightings and activation timing patterns using nonnegative matrix factorization (NNMF) described as follows:

1 $${EMG}_{o}=WH+e={EMG}_{r}+e$$

where $W$ is a $m\times n$ matrix ($n$ is the number of modules) that specifies the muscle group weightings; $H$ is a $n\times t$ matrix that represents activation timing patterns; ${EMG}_{r}$ is the reconstructed EMG composed of $m\times t$ matrix resulting from the multiplication of $W$ and $H$; $e$ is the residual error (i.e., $e={EMG}_{o}-{EMG}_{r}$). From the initialized random matrices ${W}_{i}$ and ${H}_{i}$, the NNMF algorithm iteratively updated $W$ and $H$ to minimize the residual error and searched for optimal ${EMG}_{r}$ [27, 28]. To avoid local minima depending on the initialized random matrices ${W}_{i}$ and ${H}_{i}$, we applied the algorithm 100 times and selected the initial matrices with lowest residual error [28].

The NNMF determined the minimum number of muscle modules in each leg of each subject based on a reconstruction quality criterion: variability accounted for ($VAF$) $\ge$ 90% [13]. We additionally executed the NNMF algorithm three times, considering that three to five modules were needed for the EMG reconstruction similar to the previous work [20]. In this work, the parameters to represent muscle modules included: value at $VAF$ $\ge$ 90% (${VAF}_{US}$ and ${VAF}_{AS}$), and $VAF$ values with three to five modules (${VAF}_{US,k}$ and ${VAF}_{AS,k}$, where $k=3,\cdots , 5$) because the $VAF$ is a critical information that determines number of muscle modules.

### Gait performance measure

#### Gait quality measures

We categorized the gait quality measures into spatiotemporal, limb, and joint kinematic domains [4]. The parameters of spatiotemporal and limb kinematic domains were extracted by imposing the average joint kinematics data of a single gait cycle into a lower body biomechanical model. Spatiotemporal parameters included step length ($SL$) and step time ($ST$), defined as the linear distance between right and left feet, and the duration of each step, respectively. The parameters of limb kinematics incorporate leg extension angle ($LEA$) [29], limb length ($LL$) [30], and footpath area ($FPA$) [31] defined as the angle between a line from hip to the foot and vertical before toe-off, the range of linear distance between hip and the foot, and the area under the foot pattern from hip sagittal plane during gait cycle, respectively. The parameters of joint kinematics were defined as the range of motion of selected joints including all rotations of hip, knee flexion/extension, and ankle dorsi/plantar flexion.

The symmetry index metric [9] was used to evaluate the gait quality given by

2 $${SI}_{n}=\frac{{US}_{n}-{AS}_{n}}{0.5({US}_{n}+{AS}_{n})}$$

where ${US}_{n}$ and ${AS}_{n}$ are the ${n}^{th}$ gait parameter of the unaffected and affected side, respectively, and $n$ can be the aforementioned spatiotemporal, limb and joint kinematic parameters. The value is always between − 2 to 2, and a positive (or negative) value indicates $US>AS$ (or vice versa) [9]. Note that the symmetry index, ${SI}_{n}=0$ when the gait parameter between unaffected and affected sides is in perfect symmetry (i.e., ${US}_{n}={AS}_{n}$).

#### Functional gait measure

For the functional gait measure, we selected gait speed ($GS$) because speed is a well-accepted indicator of gait performance after stroke [32].

### Statistical analysis

MATLAB (Mathworks, Inc. R2017b, Natick, MA) was used for the statistical analysis. A linear regression model was used to evaluate the relationship between gait parameters and the number of muscle modules with a significance level of $\alpha <0.05$. The dependent variables were functional measure (i.e., gait speed) and the gait quality measures including symmetry index of spatiotemporal, limb and joint kinematic parameters. The independent variable was total number of muscle modules from both sides.

Second, a stepwise multiple regression analysis was used to investigate which combination of the neuromuscular parameters was best associated with gait quality measures with a significance level of $\alpha <0.05$. A total of 24 aforementioned independent variables including the modular neuromuscular control parameters were selected (i.e., area under the EMG envelope and $VAF$). The dependent variables were the gait quality measures including symmetry index of spatiotemporal, limb and joint kinematic parameters. A preliminary analysis was conducted using a linear regression on each considered independent variable and each dependent variable to minimize the number of independent variables and to simplify the final model as possible (see Additional file 1: Table S1). Only those independent variable candidates with $p$-value $\le$ 0.05 were selected as input for the stepwise multiple regression analysis.

## Results

### Qualitative inspection of muscle activations

**Fig. 4:**
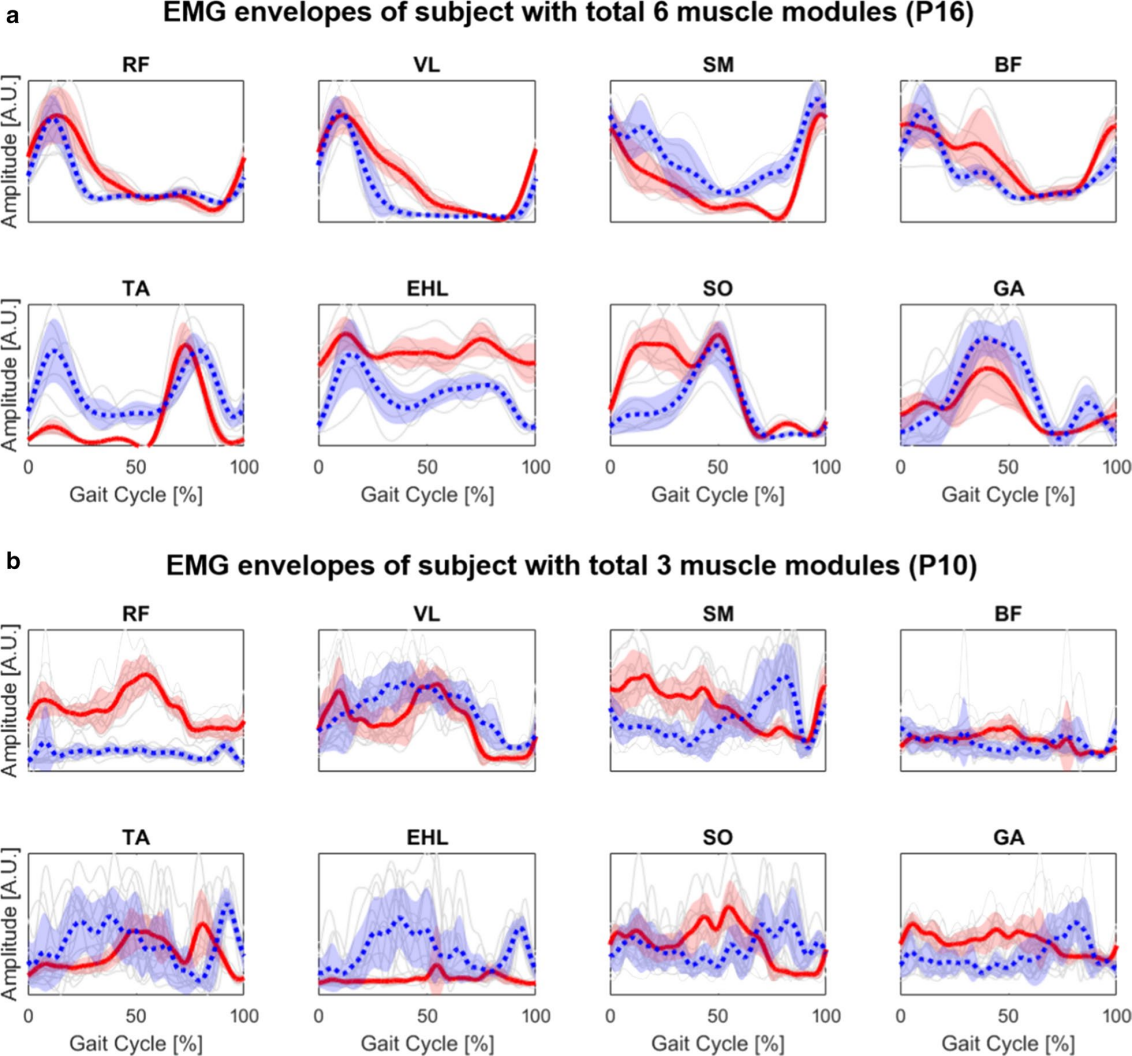
EMG envelope profiles of two subjects whose total number of modules was **a** six (P16, three modules at both sides) and **b** three (P10, two modules at unaffected side and one module at affected side). Grey lines are the EMG envelope profiles of single gait cycles. Solid red and dashed blue lines indicate average EMG envelope profiles of affected and unaffected sides, respectively, and shaded area is the standard deviation

We first observed the EMG envelope profiles of all 16 muscles of bilateral legs to qualitatively inspect overall muscle activations of post-stroke participants. In general, a large variability was observed between subjects. Figure 4 shows the EMG envelopes of two representative participants whose total number of muscle modules was six (P16, three modules at both sides) and three (P10, two modules at unaffected side and one module at affected side). Patient with six modules had relatively more smooth and consistent patterns within gait cycles compared to the patient with three modules. However, the patient with six modules showed overall more distinctive patterns whereas the patient with three modules had relatively similar and monotonic trend in EMG profiles between muscles.

### Linear regression across the total number of muscle modules

**Fig. 5:**
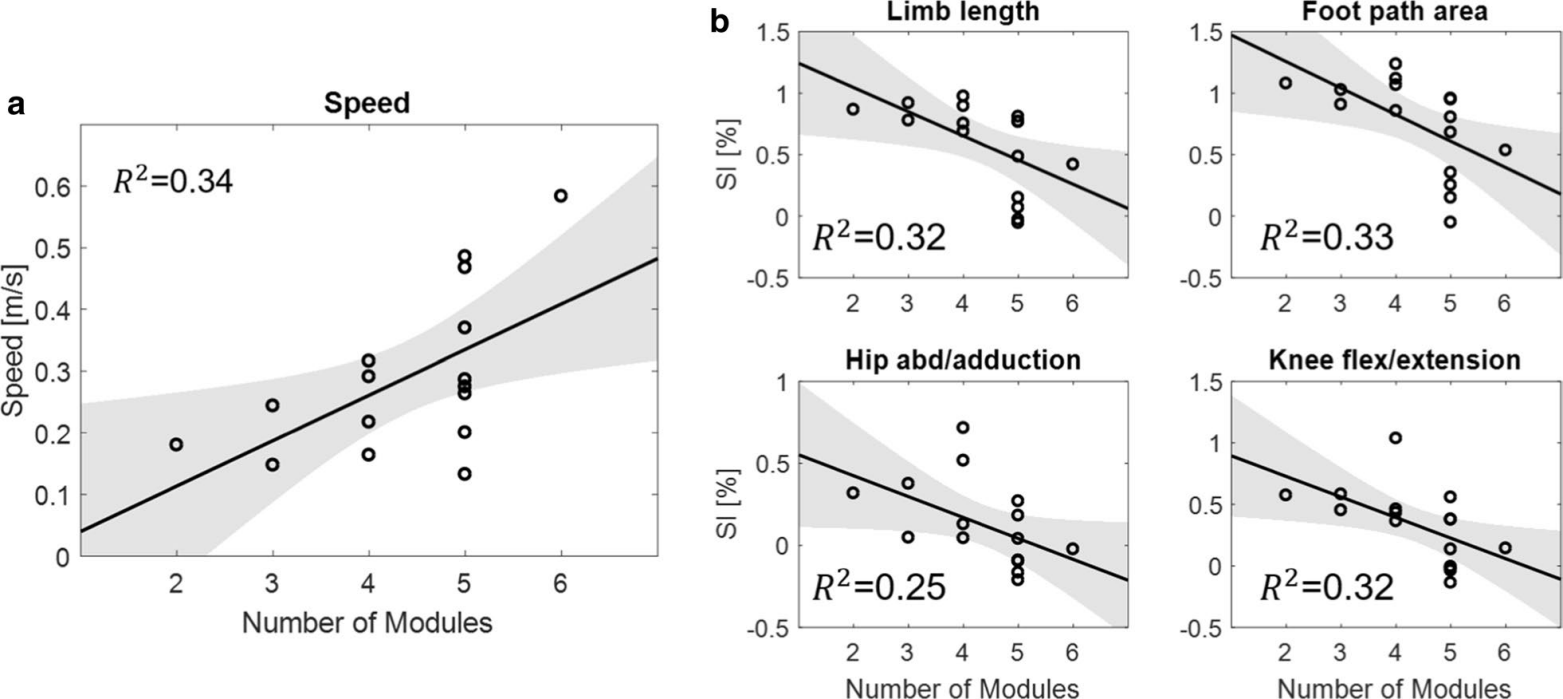
Linear regression models fitted on gait parameters, **a** Gait speed, and **b** Symmetry index ($SI$) of significant gait quality measures including limb length (top left), footpath area (top right), hip abduction/adduction (bottom left) and knee flexion/extension (bottom right) across the total number of muscle modules from both sides. The shaded area indicates 95% confidence interval

The average total number of modules was 4.37 with a minimum and maximum of two and six modules, respectively. We first fitted a linear regression model on functional gait measure (i.e., gait speed) across the total number of muscle modules. As expected, we observed a significant association between gait speed and the total number of modules ($p$ < 0.05). Among all gait quality measures, the symmetry index of $LL$ and $FPA$ from limb kinematics and hip abduction/adduction and knee flexion/extension from joint kinematics revealed a significant association with the total number of modules (all $p$ < 0.05). None of the measures from spatiotemporal characteristics revealed significant relationship with the total number of modules. Figure 5 illustrates the linear regression line fitted on gait speed (Fig. 5a) and gait quality measures with significance (Fig. 5b) across the total number of modules. All results are summarized in Table 2. These results indicate that there exists a relationship between the number of muscle modules and gait measures, suggesting that the increased muscle modules may result in improvements in gait quality and function.

**Table 2:** Results of linear regression models with symmetry index of gait quality measures (dependent variables) and the total number of muscle modules (independent variables)

Gait Features	SI Parameters [%]	$\beta$[95% CI]	F-value	p-value	${R}^{2}$ (%)	$Aadjusted {R}^{2 (\mathrm{\%})}$
Functional	Speed	0.583 [0.117, 1.048]	7.19	< 0.05	33.9	29.2
Spatiotemporal characteristics	Step length	− 0.237 [− 0.794, 0.320]	0.84	0.38	5.6	0.0
	Step time	− 0.038 [− 0.611, 0.535]	0.02	0.89	0.2	0.0
Limb kinematics	Leg extension angle	− 0.217 [− 0.776, 0.343]	0.69	0.42	4.7	0.0
	Limb length	− 0.565 [− 1.038, − 0.092]	6.56	< 0.05	31.9	27.0
	Footpath area	− 0.572 [− 1.042, − 0.102]	6.81	< 0.05	32.7	27.9
Joint kinematics (sagittal plane)	Hip flex/ex	− 0.424 [− 0.943, 0.010]	3.06	0.10	18.0	12.1
	Knee flex/ex	− 0.564 [− 1.037, − 0.090]	6.53	< 0.05	31.8	26.9
	Ankle dorsi/plantar	− 0.318 [− 0.861, 0.226]	1.57	0.23	10.1	3.7
Joint kinematics (other planes)	Hip abd/add	− 0.503 [− 0.998, − 0.007]	4.74	< 0.05	25.3	20.0
	Hip int/ext ro	0.120 [− 0.449, 0.689]	0.20	0.66	1.4	0.0

*SI* symmetry index, flex/ex flexion/extension, abd/add abduction/adduction, int/ext ro. internal/external rotation, dorsi/plantar dorsi/plantar flexion, $\beta$ slope of regression, CI confidence interval

### Stepwise regression on gait quality measures

#### Relations with spatiotemporal parameters

**Fig. 6:**
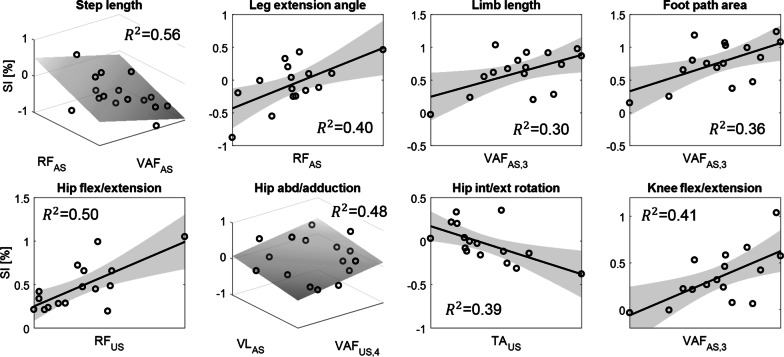
Regression models with selected independent variables (see Table 3) from stepwise multiple regression. Note that a plane in three-dimensional space is illustrated with two independent variables for step length and hip abduction/adduction

For $SL$, the stepwise regression analysis selected ${VAF}_{AS}$ (${\beta }_{1}=-0.45$) and ${RF}_{AS}$ (${\beta }_{2}=0.54$) as independent variables. The model revealed a statistically significant relationship ($F=8.26$*,* $p<0.01$) and accounted for approximately 56% of the variance of $SL$ (${R}^{2}=0.56$, $Adjusted {R}^{2}=0.49$). The visualization of the regression model with selected independent variables is shown in Fig. 6 (top left). In contrast, none of the independent variable candidates had a $p$-value $\leq0.05$ for $ST$ (see Table 3).

**Table 3:** Results of the stepwise multiple regression models with symmetry index of gait quality measures (dependent variable) and modular neuromuscular parameters (independent variable)

Gait Features	SI parameters [%]	Multiple regression model	F-value	p-value	${R}^{2}$	$Adjusted {R}^{2}$
Spatiotemporal characteristics	Step length	${SI}_{SL}=-0.45{VAF}_{AS}+0.54{RF}_{AS}$	8.26	< 0.01	56.0%	49.2%
	Step time	–	–	–	–	–
Limb kinematics	Leg extension angle	${SI}_{LEA}=0.63{RF}_{AS}$	9.16	< 0.01	39.6%	35.2%
	Limb length	${SI}_{LL}=0.55{VAF}_{AS,3}$	5.96	< 0.05	29.9%	24.8%
	Footpath area	${SI}_{FPA}=0.60{VAF}_{AS,3}$	7.73	< 0.05	35.6%	31.0%
Joint kinematics (sagittal plane)	Hip flex/ex	${SI}_{Hip,FE}=0.70{RF}_{US}$	14.0	< 0.01	50.0%	46.4%
	Knee flex/ex	${SI}_{Knee,FE}=0.64{VAF}_{AS,3}$	9.91	< 0.01	41.4%	37.3%
	Ankle dorsi/plantar	–	–	–	–	–
Joint kinematics (other planes)	Hip abd/add	${SI}_{Hip,AA}=0.48{VAF}_{US,4}+0.48{VL}_{AS}$	6.08	< 0.05	48.3%	40.4%
	Hip int/ext ro	${SI}_{Hip,IE}=-0.62{TA}_{US}$	8.93	< 0.01	38.9%	34.6%

*SI* symmetry index, *SL* step length, *LEA* leg extension angle, *LL* limb length, *FPA* footpath area, *Hip, FE* hip flexion/extension, *Hip,AA* hip abduction/adduction, *Hip,IE* hip internal/external rotation, *Knee,FE* knee flexion/extension, *VAF* variability accounted for, *RF* rectus femoris, *VL* vastus lateralis, *TA* tibialis anterior

#### Relations with limb kinematic parameters

For $LEA$, the stepwise regression analysis selected ${RF}_{AS}$ (${\beta }_{1}=0.63$) as an independent variable, was statistically significant ($F=9.16$*,* $p<0.01$), and accounted for approximately 40% of the variance (${R}^{2}=0.40$, $Adjusted {R}^{2}=0.35$). For $LL$ and $FPA$, both stepwise regression analyses selected ${VAF}_{AS,3}$ (${\beta }_{1}=0.55$ for $LL$ and ${\beta }_{1}=0.60$ for $FPA$) as an independent variable. The fitted models were statistically significant ($F=5.96$*,* $p<0.05$ for $LL$ and $F=7.73$*,* $p<0.05$ for $FPA$) and accounted for approximately 30% and 36% of the variance of $LL$ and $FPA$, respectively (${R}^{2}=0.30$, $Adjusted {R}^{2}=0.25$ for $LL$ and ${R}^{2}=0.36$, $Adjusted {R}^{2}=0.31$ for $FPA$). The results are summarized in Table 3 with the visualization of the regression models with selected independent variables shown in Fig. 6 (top right three).

#### Relations with joint kinematic parameters

For the hip flexion/extension, the stepwise regression analysis selected ${RF}_{US}$ (${\beta }_{2}=0.70$) as the independent variables and were statistically significant ($F=14.0$*,* $p<0.01$) accounting for approximately 50% of the variance (${R}^{2}=0.50$, $Adjusted {R}^{2}=0.46$). For the knee flexion/extension, the stepwise regression analysis selected $\mathrm{V}{AF}_{AS,3}$ (${\beta }_{1}=0.64$ as the independent variable. The fitted model was statistically significant ($F=9.91$*,* $p<0.01$) and accounted for approximately 41% of the variance of the knee flexion/extension (${R}^{2}=0.41$, $Adjusted {R}^{2}=0.37$). For the hip abduction/adduction, the stepwise regression analysis selected ${VAF}_{US,4}$ (${\beta }_{1}=0.48$) and ${VL}_{AS}$ (${\beta }_{2}=0.48$) as the independent variables and were statistically significant ($F=6.08$*,* $p<0.05$) accounting for approximately 48% of the variance (${R}^{2}=0.48$, $Adjusted {R}^{2}=0.40$). For the hip int/external rotation, the stepwise regression analysis selected ${TA}_{US}$ (${\beta }_{1}=-0.62$) as the independent variable and was statistically significant ($F=8.93$*,* $p<0.01$) accounting for approximately 39% of the variance (${R}^{2}=0.39$, $Adjusted {R}^{2}=0.35$). Finally, for the ankle dorsi/plantar flexion, none of the independent variable candidates had a $p$-value $\leq0.05$ (see Table 3). The visualization of the regression models with selected independent variables is shown in Fig. 6 (bottom row).

Overall, these results indicate that there exists a significant relationship between the neuromuscular control parameters and the gait quality measures.

## Discussion

The primary goal of this study was to explore the relations of modular neuromuscular control parameters and quality of movement during gait after stroke. The main findings were as follows: first, post-stroke individuals with reduced number of muscle modules exhibited worse gait function and greater asymmetry in gait quality measures, particularly in kinematics level. Second, the gait quality measures revealed a significant correlation with modular neuromuscular control parameters extracted from the EMG data. Specifically, those parameters were variability accounted for ($VAF$) information from muscle modules (i.e., ${VAF}_{AS}$, ${VAF}_{AS,3}$, and ${VAF}_{US,4}$) and area under the EMG envelope curves from rectus femoris, vastus lateralis, and tibialis anterior muscles (i.e., ${RF}_{AS}$, ${RF}_{US}$, ${VL}_{AS}$, and ${TA}_{US}$). To our knowledge, this work is novel in the fact that it integrates detailed gait quality measures with the neuromuscular control framework. The results in this study offer preliminary evidence justifying that the modular neuromuscular framework can be a useful indicator of gait quality measures and help to understand the underlying mechanism of how gait quality is disturbed after stroke.

The analysis of the muscle modules has become a more popular tool to describe the neuromotor control of multi-limb movement such as complicated tasks that require proficiency or gait after stroke [13, 33]. For the analysis, many previous studies classified muscle modules into paretic and non-paretic sides [13, 20, 34]. While this is a reasonable separation given that most stroke populations exhibit a hemiplegic gait, our rationale was that walking is essentially the performance of an inter-coordinated behavior between both legs [15, 35]. Thus, we analyzed the data from a different perspective, combining the number of muscle modules of both sides, and observed more strong trends than using affected or unaffected side alone (see $p$-values in Additional file 1: Table S2). Our data revealed that there is a significant, positive correlation between gait quality measures and number of modules, indicating better gait quality with greater number of muscle modules. This result is consistent with a previous study that found merged muscle module is critical to poor walking performance [34]. This also justifies the investigation of therapeutic interventions that can increase the number of modules to improve the gait quality after stroke [17].

One thing to note is that most participants in our data had a relatively severe gait impairment with slow walking speed (average speed: 0.29 $\pm$ 0.13 m/s, see Table 1) [36]. Nevertheless, we found significant trend in gait quality measures across the number of muscle modules, mostly observed in the kinematic parameters (see Table 2). However, none of the parameters from spatiotemporal characteristics revealed a significant trend. These results correspond with previous research that found post-stroke individuals exhibit significant asymmetry in joint kinematics with greater inter-individual variability than spatiotemporal characteristics [10]. Given that significance was observed within a severe population in our data, we speculate to find a more distinct trend with a larger sample size with various severity levels including mild to moderately impaired stroke population.

A recent study attempted to combine parameters from muscle modules and gait analysis (i.e., spatiotemporal and joint kinematics) to predict functional outcomes such as walking speed [20]. While this study successfully predicted locomotor function with combined biomechanical and neuromuscular measures, the relations between these measures, which would more likely to have direct causal relations, were not analyzed. Thus, we used the stepwise multiple regression approach to find the optimal linear regression model that correlates the gait quality measures with the input modular neuromuscular control parameters. We found that all symmetry indices of gait quality measures, except for step time and ankle dorsi/plantar flexion, were linked with the neuromuscular modular control parameters with significant association (see Table 3). Common predictors included quadriceps muscles (i.e., rectus femoris and vastus lateralis) and information from variability accounted for ($VAF$) in the regression models. The inclusion of quadriceps were expected as these muscles have long been believed to be key contributors to hip and knee motion during gait [37]. $VAF$s were also commonly selected because these parameters are critical information determining the number of muscle modules [20]. On the other hand, it is unclear why the tibialis anterior muscle appeared to be negatively associated with hip internal/external rotation given that this muscle is known as ankle dorsiflexor. This result may be related to abnormal compensatory coupling between irrelevant muscles due to neurological impairment [38]. However, further research is needed where the EMG measures at other locations such as hip lateral rotator group muscles are added to investigate this connection, which may indeed be epiphenomenal. Overall, these results suggest a relationship exists between the neuromuscular control framework and the gait quality measures.

One possible therapeutic application of this connection would be using the concept of neuromuscular and gait quality parameters as outcome measures to assess gait impairment after stroke. An example of previous research would be a locomotor intervention study that applied muscle modules as pre- and post-therapy outcome measures [17, 39]. These studies found increase in number of muscle modules and improvements in quality of modular organization (i.e., timing and compositions) as well as clinical measures such as gait speed after a locomotor rehabilitation therapy. This indicates that therapeutic interventions can change or improve the neuromuscular control framework, but the influence of these interventions on detailed movement quality was not reported in these studies. Thus, we expect the application of the neuromuscular and gait quality parameters as outcome measures will fill the gap and provide a more comprehensive characterization of gait recovery post-stroke.

The purpose of this proof-of-concept study was to provide an initial evidence of association between gait quality and neuromuscular parameters for chronic post-stroke individuals. However, this study was limited to a small sample size of 16 patients with a restricted range of impairment level; only severely impaired individuals with a slow walking speed participated in this work. However, our data still showed clear initial results on the relative importance of several predictors, providing the significant relationships exist between neuromuscular control framework and gait quality measures. We expect this methodology can be potentially used for gait training research or in clinical practice to better understand impairments related to gait function.

Although we found significant correlations in our models, there was a limitation of interpretability within the selected input modular neuromuscular control parameters. For example, while we have identified $VAF$ as a significant predictor in several regression models, it may be difficult to explain the physical meaning of the model due to the lack of physiological meaning in $VAF$. Nevertheless, we included $VAF$ s as our modular neuromuscular framework because $VAF$ comprises an important information that determines the number of muscle modules by evaluating similarity between the reconstructed EMG signals from the muscle modules compared to the original EMG signals [20]. In addition, we only included area under the envelope curve to represent EMG signal characteristics of individual muscles. However, it is possible that there might exist other EMG features [24, 40] that have more strong relations with the gait quality measures used in this work. Another related limitation includes the selection of EMG locations only in the major sagittal plane motion, which limited our interpretation on disturbed gait quality in other planes. The number of tested EMGs (i.e., 16 channels in this work) may also have influenced our results as the number of muscle modules can be increased with more tested muscles. Future work could incorporate additional EMG features including other major muscles with non-sagittal plane motion to better understand the mechanism of the impaired neuromuscular control of gait after stroke.

## Conclusions

The purpose of this study was to investigate the influence of modular neuromuscular control framework on gait quality measures. We observed that chronic post-stroke individuals with a lower number of muscle modules exhibit reduced gait function with greater deficit in gait quality measures, particularly in kinematics level. We also found that there exists a significant relationship between the neuromuscular control framework and gait quality measures. These promising results justify further research with a larger post-stroke population and expanded range of impairment level for a more reliable generalization.

## Supplementary Information

**Additional file 1**: **Table S1**. Independent variable candidates from preliminary analysis using linear regression on each considered independent variable and each dependent variable. **Table S2**. Results of linear regression models with symmetry index of gait quality measures (dependent variables) and the number of muscle modules at unaffected and affected side (independent variables).

## Data Availability

The data collected in this study are available from the corresponding author on reasonable request.

## Abbreviations

AS: Affected side
BF: Biceps femoris
EHL: Extensor halluces longus
EMG: Electromyography
FPA: Footpath area
GA: Gastrocnemius
GS: Gait speed
LEA: Leg extension angle
LL: Limb length
NNMF: Nonnegative matrix factorization
RF: Rectus femoris
SI: Symmetry index
SL: Step length
SM: Semitendinosus
SO: Soleus
ST: Step time
TA: Tibialis anterior
US: Unaffected side
VL: Vastus lateralis
VAF: Variability accounted for
