# Supplementary material for: Relationship between gait quality measures and modular neuromuscular control parameters in chronic post-stroke individuals
Source: J Neuroeng Rehabil. 2021 Apr 7;18:58. doi: 10.1186/s12984-021-00860-0 (PMC8028248; doi:10.1186/s12984-021-00860-0)
Supplement: Supplementary file 1 — Additional file 1: Table S1. Independent variable candidates from preliminary analysis using linear regression on each considered independent variable and each dependent variable. Table S2. Results of linear regression models with symmetry index of gait quality measures (dependent variables) and the number of muscle modules at unaffected and affected side (independent variables). [file 12984_2021_860_MOESM1_ESM.docx]

**Additional file 1: Table S1.** Independent variable candidates from preliminary analysis using linear regression on each considered independent variable and each dependent variable

| Gait Features | SI parameters [%] | Independent variable candidates |
| --- | --- | --- |
| Spatiotemporal characteristics | Step length | ${VAF}_{AS}$, ${RF}_{AS}$ |
|  | Step time | - |
| Limb kinematics | Leg extension angle | ${RF}_{AS}$ |
|  | Limb length | ${VAF}_{AS,3}$ |
|  | Footpath area | ${VAF}_{AS,3}$ |
| Joint kinematics (sagittal plane) | Hip flex/ex | ${VAF}_{AS,3}$, ${RF}_{US}$ |
|  | Knee flex/ex | ${VAF}_{AS,3}$ |
|  | Ankle dorsi/plantar | - |
| Joint kinematics (other planes) | Hip abd/add | ${VAF}_{US,4}$, ${VL}_{AS}$ |
|  | Hip int/ext ro. | ${TA}_{US}$ |

SI – symmetry index, flex/ex – flexion/extension, abd/add – abduction/adduction, int/ext ro. – internal/external rotation, dorsi/plantar – dorsi/plantar flexion, *VAF* – variability accounted for, *AS* – affected side, *US* – unaffected side, *RF* – rectus femoris, *GS* – gait speed, *VL* – vastus lateralis, *TA* – tibialis anterior

**Additional file 1: Table S2.** Results of linear regression models with symmetry index of gait quality measures (dependent variables) and the number of muscle modules at unaffected and affected side (independent variables)

| Gait Features | SI Parameters [%] | Unaffected side | | Affected side | |
| --- | --- | --- | --- | --- | --- |
|  |  | $\beta$ [95% CI] | p-value | $\beta$ [95% CI] | p-value |
| Functional | Speed | 0.539 [0.057, 1.022] | < 0.05 | 0.449 [-0.063, 0.961] | 0.08 |
| Spatiotemporal characteristics | Step length | -0.00 [-0.577, 0.570] | 0.99 | -0.420 [-0.940, 0.100] | 0.11 |
|  | Step time | -0.045 [-0.528, 0.617] | 0.87 | -0.117 [-0.686, 0.452] | 0.67 |
| Limb kinematics | Leg extension angle | 0.008 [-0.565, 0.581] | 0.98 | 0.396 [-0.922, 0.131] | 0.13 |
|  | Limb length | -0.447 [-0.960, 0.066] | 0.08 | -0.519 [-1.009, -0.029] | < 0.05 |
|  | Footpath area | -0.497 [-0.995, -0.000] | 0.05 | -0.476 [-0.980, -0.028] | 0.06 |
| Joint kinematics (sagittal plane) | Hip flex/ex | -0.431 [-0.948, 0.087] | 0.10 | -0.284 [-0.834, 0.265] | 0.29 |
|  | Knee flex/ex | -0.470 [-0.976, 0.036] | 0.07 | -0.382 [-0.912, 0.147] | 0.14 |
|  | Ankle dorsi/plantar | 0.130 [-0.439, 0.698] | 0.63 | 0.072 [-0.500, 0.644] | 0.79 |
| Joint kinematics (other planes) | Hip abd/add | -0.509 [-1.003, -0.016] | < 0.05 | -0.449 [-0.961, 0.064] | 0.08 |
|  | Hip int/ext ro. | -0.304 [-0.850, 0.242] | 0.25 | -0.234 [-0.791, 0.323] | 0.38 |

SI – symmetry index, flex/ex – flexion/extension, abd/add – abduction/adduction, int/ext ro. – internal/external rotation, dorsi/plantar – dorsi/plantar flexion, $\beta$ – slope of regression, CI – confidence interval
